# Complexes With Biologically Active Ligands. Part 11^1^. Synthesis and Carbonic Anhydrase Inhibitory Activity of Metal Complexes of 4,5-Disubstituted-3-Mercapto-1,2,4-Triazole Derivatives

**DOI:** 10.1155/MBD.1998.11

**Published:** 1998

**Authors:** Andrea Scozzafava, Christine Cavazza, Claudiu T. Supuran, Ioana Saramet, Fabrizio Briganti, Mircea D. Banciu

**Affiliations:** 1 Università degli Studi Dipartimento di Chimica Laboratorio di Chimica Inorganica e Bioinorganica Via Gino Capponi 7 Florence I-50121 Italy; 2 Faculty of Pharmacy Department of Chemistry T. Vuia Str., 6 Bucharest Romania; 3 Polytechnic University Department of Organic Chemistry Splaiul Independentei 313 Bucharest Romania

## Abstract

Complexes containing five 4,5-disubstituted-3-mercapto-1,2,4-triazoles and Zn(II), Hg(II) and Cu(I) were synthesized and characterized by standard procedures (elemental analysis; IR, electronic and NMR spectroscopy, conductimetry and TG analysis). Both the thione as well as the thiolate forms of the ligands were evidenced to interact with the metal ions in the prepared complexes. The original mercaptans and their metal complexes behave as inhibitors of three carbonic anhydrase (CA) isozymes, CA I, II and IV, but did not lower intraocular pressure in rabbits in animal models of glaucoma.


### COMPLEXES WITH BIOLOGICALLY ACTIVE LIGANDS. Part 11[1]. SYNTHESIS AND CARBONIC ANHYDRASE INHIBITORY ACTIVITY OF METAL COMPLEXES OF 4,5-DISUBSTITUTED-3-MERCAPTO-1,2,4-TRIAZOLE DERIVATIVES

Andrea Scozzafava[1], Christine Cavazza[1], Claudiu T. Supuran[1*], Ioana Saramet[2], Fabrizio Briganti[1] and Mircea D. Banciu[3]

[1] Università degli Studi, Dipartimento di Chimica, Laboratorio di Chimica Inorganica e Bioinorganica, Via Gino Capponi 7, I-50121, Florence, Italy
[2] Faculty of Pharmacy, Department of Chemistry, T. Vuia Str., 6, Bucharest, Roumania
[3] Polytechnic University, Department of Organic Chemistry, Splaiul Independentei 313, Bucharest, Roumania

**Abstract**: Complexes containing five 4,5-disubstituted-3-mercapto-1,2,4-triazoles and Zn(II), Hg(II) and Cu(I) were synthesized and characterized by standard procedures (elemental analysis; IR, electronic and NMR spectroscopy, conductimetry and TG analysis). Both the thione as well as the thiolate forms of the ligands were evidenced to interact with the metal ions in the prepared complexes. The original mercaptans and their metal complexes behave as inhibitors of three carbonic anhydrase (CA) isozymes, CA I, II and IV, but did not lower intraocular pressure in rabbits in animal models of glaucoma.

### Introduction

Carbonic anhydrase (CA, EC 4.2.1.1), an enzyme playing a central role to both transport and metabolic processes involving $CO_2$ and bicarbonate, is present in a variety of tissues of higher vertebrates in the form of eight isozymes [2-4]. By catalyzing the reversible interconversion between the two chemical species mentioned above, in metabolically active tissues (such as the muscle), cytoplasmic (CA I-III) and sarcolemmal (CA III) isozymes facilitate $CO_2$ transport out of the cell [3]. The only membrane-bound isozyme known (CA IV), which is highly abundant in the kidneys and lungs, has been shown to possess an extracellular orientation of the active site, and to be critical in acidifying the outer boundary layer through the protons formed by $CO_2$ hydration [5,6], a process that then facilitates among others cellular ammonia transport by providing the $H^+$ ion for the protonation of $NH_3$ and maintaining thus the trans-membrane ammonia gradient [3,5].

The mitochondrial isozyme (CA V) is known to supply bicarbonate/$CO_2$ for the initial reaction of gluconeogenesis and ureagenesis in many mammalian tissues [7,8], as well as for the pyruvate carboxylation in the *de novo* lipogenesis in adipocytes [9].

Aromatic/heterocyclic sulfonamides with the general formula R-$SO_2NH_2$ act as powerful inhibitors of the above mentioned isozymes [4, 10-16], although relevant differences of affinity for these inhibitors between them have been evidenced [4]. Thus, CA II is the most susceptible to inhibition by sulfonamides [4], followed by CA IV and V [6, 9], whereas CA I has generally a lower affinity for this type of inhibitors and a much larger one (as compared to the previously mentioned isozymes) for the inorganic complexing anions, such as cyanide, cyanate, thiocyanate [4,17-19]. Endly, CA III is a sulfonamide-resistant isozyme [20], being appreciably inhibited only at very large (millimolar) concentrations of inhibitor (the other isozymes may be inhibited at micromolar - nanomolar concentrations of sulfonamides such as acetazolamide **1**, benzolamide **2**, ethoxzolamide **3**, dichlorophenamide **4** or dorzolamide **5** - all clinically used drugs [21-24]). The main application of such agents is as antiglaucoma drugs [22-24], but they are also used as antiulcer [21], diuretic [25], or antiepileptic drugs [26], as well as diagnostic tools in NMR imaging [27, 28]. Although many sulfonamide CA inhibitors possess high affinity for the major isozymes considered to play important physiological functions (such as CA II, CA IV and CA V) [4, 7, 8, 11-16], the critical challenge for the design of novel pharmacological agents from this class, is constituted by the lack of specificity of such compounds towards the different isozymes [4, 29, 30]. Thus, the search of compounds from other classes

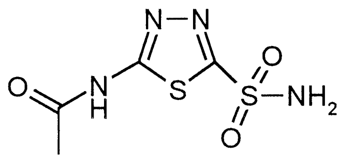

**1**

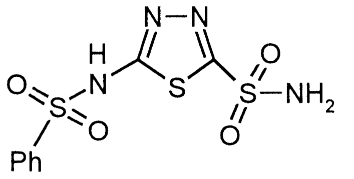

**2**

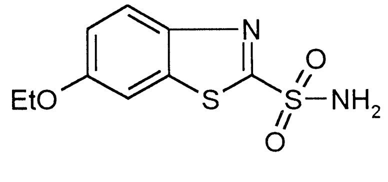

**3**

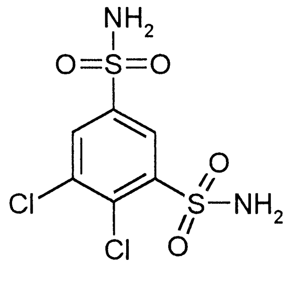

**4**

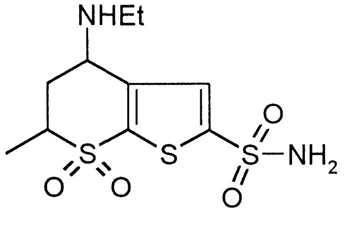

**5**

than the sulfonamides, which might act as powerful inhibitors of different CA isozymes, is actively involving several laboratories, and interesting results have emerged recently [31 – 35]. Recently, Christianson's group [35] proved that hydroxamate **6** acts as a potent inhibitor of CA II, whereas we have investigated the interaction of isozymes I and II with mercaptans **7** and **8**, and some of their metal complexes [34, 36].

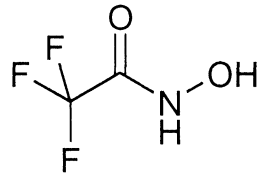

**6**

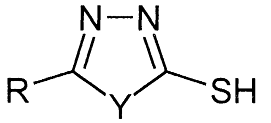

**7**

**a: R = $H_2N$; Y = S**

**b: R = AcNH; Y = S**

**c: R = $H_2N$; Y = NH**

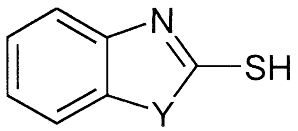

**8**

**a: Y = S**
**b: Y = NH**

The powerful inhibition observed with some heterocyclic mercaptans of type **7** and **8** [34, 36] prompted us to extend researches in this class of compounds. In this paper we report inhibition studies against CA isozymes I, II and IV with five 4,5-disubstituted-3-mercapto-1,2,4-triazoles and their Zn(II), Hg(II) and Cu(I) complexes, which were synthesized and characterized by standard procedures.

### Materials and Methods

IR spectra were recorded on a Perkin-Elmer 16PC FTIR instrument, in the range 200-4000 $cm^{-1}$, in KBr pellets. Solution electronic spectra were recorded with a Cary 3 spectrophotometer interfaced with a PC. Conductimetric measurements were done in DMF solutions, at 25°C (concentrations of 1 mM of complex) with a Fisher conductimeter. Magnetic susceptibility measurements were carried out at room temperature with a fully automated AZTEC DSM8 pendulum-type susceptometer. Mercury(II) tetrakis-(thiocyanato)cobaltate(II) was used as a susceptibility standard. Corrections for the diamagnetism were estimated from Pascal's constants [37]. Elemental analyses were done by combustion for C, H, N with an automated Carlo Erba analyzer, and gravimetrically for the metal ions, and were ± 0.4% of the theoretical values. NMR spectra were recorded in DMSO-$d_6$ as solvent with a Bruker CPX-200 instrument. Thermogravimetric measurements were done in air, at a heating rate of 10°C/min., with a Perkin Elmer 3600 thermobalance.

Mercaptans **9-13** were prepared as described in the literature [38]. Metal salts (Cu(I) chloride, Hg(II) chloride and Zn(II) sulfate pentahydrate), triethylamine and solvents were from E. Merck (analytical grade) and were used without additional purification.

Human CA I and CA II cDNAs were expressed in *Escherichia coli* strain BL21 (DE3) from the plasmids pACA/HCA I and pACA/HCA II (the two plasmids were a gift from Prof. Sven Lindskog, Umea University, Sweden). Cell growth conditions were those described by Lindskog's group [39] and enzymes were purified by affinity chromatography according to the method of Khalifah et al. [40]. Enzyme concentrations were determined spectrophotometrically at 280 nm, using a molar absorptivity of 49 $mM^{-1}.cm^{-1}$ for CA I and 54 $mM^{-1}.cm^{-1}$ for CA II, respectively, based on $M_r$ = 28.85 kDa for CA I, and 29.3 kDa for CA II, respectively [41]. CA IV was isolated from bovine lung microsomes [42].

Initial rates of 4-nitrophenyl acetate hydrolysis were monitored spectrophotometrically, at 400 nm and 25°C, with a Cary 3 apparatus interfaced with an IBM compatible PC by the method of Pocker and Stone [43]. Solutions of substrate were prepared in anhydrous acetonitrile; the substrate concentrations varied between $10^{-2}$ and $10^{-6}$ M. A molar absorption coefficient $\varepsilon$ = 18,400 $M^{-1}.cm^{-1}$ was used for the 4-nitrophenolate formed by hydrolysis, in the conditions of the experiments (pH 7.80), as reported by Pocker and Stone [43]. Non-enzymatic hydrolysis rates were always subtracted from the observed rates. Duplicate experiments were done for each inhibitor, and the values reported throughout the paper are the averages of such results. $IC_{50}$ represents the molarity of inhibitor producing a 50% decrease of enzyme catalyzed hydrolysis of 4-nitrophenyl acetate.

**General procedure for the preparation of the Zn(II) and Hg(II) complexes 14-23**

4 mMol of mercaptan **9-13** were suspended in 25 mL MeOH and 4mMol of $Et_3N$ were added in order to deprotonate the ligand. This was treated thereafter with a methanolic solution of the metal salt (Zn(II) sulfate and Hg(II) chloride, respectively), working at the molar ratios $M^{2+}$ : mercaptan of 1:2. A yellowish-white precipitate formed immediately. The obtained reaction mixture was heated on a steam bath for 2 hours, then the precipitated complexes were filtered, thoroughly washed with cold alcohol and air dried. Crystallization was not done as the only solvents in which the complexes possessed good solubility were DMSO and DMF. The obtained powders of complexes **14-23** melted with decomposition at temperatures higher than 300 °C.

**Preparation of the Cu(I) complexes 24-28**

An amount of 6 mMol of the ligand 9-13 in 50 mL of absolute ethanol was added to 0.300 g CuCl (3 mMol) dissolved in 30 mL of anhydrous acetonitrile. The gelatinous cream-like precipitate was filtrated with suction and air dried under reduced pressure over silica gel at room temperature. Basically this method used by us is the same as the one described by Raper's group [44-46] for the preparation of Cu(I) complexes of imidazole thiones and related ligands.

**Results and Discussion**

The heterocyclic mercaptans **9-13** used for the preparation of metal complexes, were deprotonated in the presence of triethylamine prior to complexation with Zn(II) and Hg(II),or were used as neutral ligands in

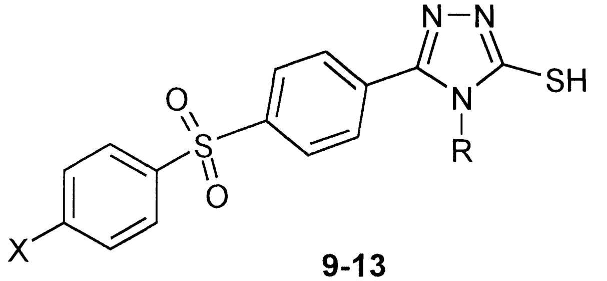

**9-13**

| | X | R |
|---|---|---|
| **9** : | H | Et |
| **10**: | Cl | Et |
| **11**: | Br | Et |
| **12**: | Cl | $C_6H_{11}$ |
| **13**: | Cl | H |

the case of the Cu(I) derivatives, as described by Raper's group for similar ligands [44-46].

One of the main complication which one has to face when preparing metal complexes of ligands of the type used by us in the present work, regards the many tautomeric forms of such compounds [44-46]. As for the related systems of 1-methylimidazoline-2(3H)-thione, thoroughly investigated by Raper's group [44-46] or the 4-amino-1,4-dihydro-3-methyl-1,2,4-triazole-5-thione investigated by Pellinghelli's group [47], compounds **9-13** exhibit the thiol – thione tautomerism presented in Scheme 1.

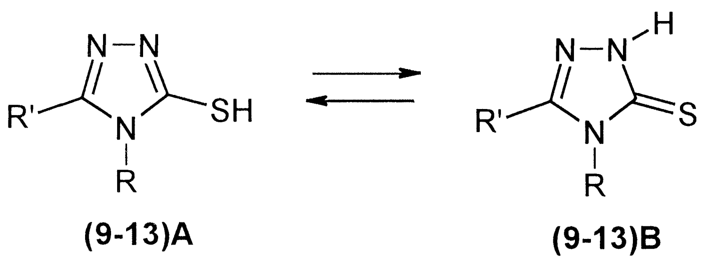

**Scheme 1**

In the solid state and in neutral solution, the thione forms of type **B** are the dominant tautomers, with the thione sulphur atom as the favoured donor site [44-46]. Deprotonation of these derivatives in a variety of conditions generates thionate anions in which both the thionate sulphur as well as the endocyclic nitrogen atoms are available, either singly or collectively, for coordination. In this work we have used both these possibilities for preparing metal complexes of derivatives **9-13**. Thus, in the case of the Zn(II) and Hg(II) derivatives, the metal complexes were prepared using the deprotonated derivatives **9-13**, whereas the Cu(I) complexes were obtained from the neutral (thione) form of the ligands, as in the classical studies of Raper's group [44-46].

The new complexes **14-28** reported in the present work and their elemental analysis data are shown in Table I.

Table I: Prepared complexes **14-28**, containing the mercapto-triazoles **9 - 13** (LH) or their conjugate bases (L) as ligands and their elemental analysis data.

| No. | Complex | Ligand LH | %M[a] | %C[b] | %H[b] | %N[b] |
|---|---|---|---|---|---|---|
| | | | Analysis (calculated/found)* | | | |
| **14** | $[ZnL_2]$ | **9** | 8.6/8.5 | 50.9/51.1 | 3.7/3.3 | 11.1/10.9 |
| **15** | $[ZnL_2]$ | **10** | 7.9/8.1 | 46.6/46.4 | 3.1/3.3 | 10.2/10.0 |
| **16** | $[ZnL_2]$ | **11** | 7.1/6.9 | 42.1/41.8 | 2.8/2.7 | 9.2/9.0 |
| **17** | $[ZnL_2]$ | **12** | 7.0/7.0 | 51.5/51.5 | 4.0/4.3 | 9.0/8.8 |
| **18** | $[ZnL_2]$ | **13** | 8.5/8.1 | 43.8/43.5 | 2.3/2.2 | 10.9/10.8 |
| **19** | $[HgL_2]$ | **9** | 22.5/22.2 | 43.1/43.1 | 3.1/2.8 | 9.4/9.4 |
| **20** | $[HgL_2]$ | **10** | 20.9/20.7 | 40.0/40.1 | 2.7/2.3 | 8.7/8.5 |
| **21** | $[HgL_2]$ | **11** | 19.1/19.1 | 36.6/36.2 | 2.4/2.5 | 8.0/7.9 |
| **22** | $[HgL_2]$ | **12** | 18.8/18.6 | 45.0/45.2 | 3.5/3.5 | 7.8/7.5 |
| **23** | $[HgL_2]$ | **13** | 22.2/22.4 | 37.2/37.0 | 1.9/1.7 | 9.3/9.0 |
| **24** | $[Cu(LH)_2Cl]$ | **9** | 8.0/8.1 | 48.6/48.9 | 3.8/3.7 | 10.6/10.3 |
| **25** | $[Cu(LH)_2Cl]$ | **10** | 7.4/7.5 | 44.7/44.3 | 3.2/3.2 | 9.7/9.6 |
| **26** | $[Cu(LH)_2Cl]$ | **11** | 6.7/6.6 | 40.5/40.1 | 2.9/2.6 | 8.8/8.5 |
| **27** | $[Cu(LH)_2Cl]$ | **12** | 6.5/6.7 | 49.6/49.2 | 4.1/4.3 | 8.6/8.2 |
| **28** | $[Cu(LH)_2Cl]$ | **13** | 7.9/7.5 | 41.8/41.7 | 2.5/2.2 | 10.4/10.0 |

[a]By gravimetry; [b]By combustion; * No weight loss observed under 200 °C by TG analysis.

The most important IR bands in the spectra of compounds **9-28** are shown in Table II. Several important features of these spectra should be mentioned: (i) the two $SO_2$ vibrations appear unchanged in the IR spectra of the ligands **9-13** and their metal complexes **14-28**, proving that these moieties do not interact with the metal ions (data not shown); (ii) major modifications in the spectra of the complexes as compared to those of the corresponding ligands, regard the thioamide vibrations (Table II). Thus, with the exception of the thioamide III band, generally appearing at the same wavelength in the spectra of the ligands and those of the complexes, the other three thioamide bands are perturbed by the presence of the metal ions. In the spectra of complexes, the thioamide II and IV bands appeared with 5 – 45 $cm^{-1}$ at lower wavelength as compared to the corresponding band of the ligand, whereas the thioamide I band appeared with 20-30 $cm^{-1}$ at higher wavelength for the Zn(II) and Hg(II) complexes, and were unchanged for the Cu(I) derivatives. The most perturbed was the thioamide IV band, which was generally splitted in two or three intense bands in the

Table II: IR bands and their assignement for derivatives **9-28**.

| Compounds | IR band ($cm^{-1}$) | | | | | |
|---|---|---|---|---|---|---|
| | ν(NH) | Thioamide I | Thioamide II | Thioamide III | Thioamide IV | ν(M-X)[a] |
| **9** | 3090 | 1480 | 1280 | 1090 | 770 | - |
| **14** | - | 1500 | 1275 | 1090 | 750, 800 | 440, 590 |
| **19** | - | 1500 | 1280 | 1090 | 740, 800 | 415, 540 |
| **24** | 3090 | 1480 | 1270, 1285 | 1090 | 730, 745, 790 | - |
| **10** | 3085 | 1470 | 1275 | 1090 | 775 | - |
| **15** | - | 1500 | 1270 | 1090 | 740, 790 | 435, 590 |
| **20** | - | 1500 | 1270 | 1090 | 730, 800 | 415, 545 |
| **25** | 3085 | 1470 | 1260, 1280 | 1090 | 725, 750, 785 | - |
| **11** | 3080 | 1470 | 1280 | 1075 | 760 | - |
| **16** | - | 1500 | 1270 | 1075 | 745, 780 | 430, 600 |
| **21** | - | 1495 | 1270 | 1070 | 750, 780 | 410, 550 |
| **26** | 3080 | 1470 | 1270, 1285 | 1080 | 730, 745, 775 | - |
| **12** | 3090 | 1475 | 1280 | 1085 | 775 | - |
| **17** | - | 1500 | 1260 | 1080 | 735, 790 | 440, 580 |
| **22** | - | 1500 | 1260 | 1085 | 730, 780 | 410, 550 |
| **27** | 3090 | 1475 | 1265, 1290 | 1080 | 730, 750, 785 | - |
| **13** | 3290 | 1470 | 1280 | 1080 | 770 | - |
| **18** | 3260 | 1500 | 1275 | 1080 | 725, 785 | 445, 600 |
| **23** | 3260 | 1500 | 1270 | 1080 | 725, 790 | 415, 540 |
| **28** | 3290 | 1470 | 1260, 1285 | 1080 | 725, 755, 790 | - |

[a] M-S or M-N vibrations.

spectra of all metal complexes, whereas in the spectra of the ligands it was presented as a single band. This behaviour has been previusly reported for other metal complexes of heterocyclic thiones [44-46]; (iii) a clear distinction could be made regarding the tautomeric form of the ligand in the case of the Zn(II) and Hg(II) complexes on one hand, and the Cu(I) derivatives on the other hand. Thus, for the first derivatives (prepared from the deprotonated form of the ligand) no NH bands were evidenced in the IR spectra (these bands were present in the spectrum of the corresponding ligand), whereas for the Cu(I) derivatives these are present at the same frequencies as in the case of the ligand. The thioamide I band is also different for the two groups of complexes, with the Cu(I) derivatives showing this band at the same frequency as in the case of the ligand, whereas for the Zn(II) and Hg(II) derivatives the correponding band was shifted at higher wavelength. The thioamide II band on the other hand was splitted only in the case of the Cu(I) derivatives. In the $^{1}$H- and $^{13}$C-NMR data of the Hg(II) complexes, the following modifications have been noted as compared to the corresponding spectra of the original mercaptans **9-13**: (i) the signal of the NH proton, appearing at 8.30 – 8.35 ppm in the $^{1}$H-NMR spectra of compounds **9-13**, is absent in the spectra of the corresponding Zn(II) and Hg(II) complexes, **14-23**; (ii) other signals (the aromatic protons, the moiety substituting the N-4 atom) appear in the same ranges in the original mercaptans and the corresponding Zn(II) and Hg(II) complexes (data not shown); (iii) in the $^{13}$C-NMR spectra, the C-3 carbon atoms show a signal at 168.2 – 168.6 ppm in the spectra of compounds **9-13**, whereas in the spectra of the corresponding Zn(II) and Hg(II) complexes these signals appear at 165-167 ppm. This shift is presumably due to the presence of the coordinated metal ions in the neighbourhood of these atoms; (iv) other signals in the $^{13}$C-NMR spectra of mercaptans **9-13** and metal complexes **14-23** appear unchanged, at the same chemical shifts (data not shown). All the prepared complexes showed no weight loss under 200°C, were non-electrolytes (in DMF as solvent, at room temperature – data not shown), diamagnetic (data not shown) and colorless also in the case of the Cu(I) derivatives, prompting us to propose the structures shown below. All these data indicate that in the case of the Zn(II) and Hg(II) derivatives the ligand acts in deprotonated form, bidentately, with the donor system probably constituted by the endocyclic nitrogen and the mercaptide sulfur atoms. In the case of the Cu(I) derivatives, the thione form of the ligands probably acts monodentately, with the thione sulfur as donor atom, similarly as in the Cu(I) derivatives of 1-methylimidazoline-2(3H)-thione reported by Raper's group [44-46]. The Cu(I) derivatives are probably dimers, as the similar complexes reported by Raper's group [44-46].

Inhibition data against three CA isozymes, CA I, II (cytosolic) and IV (membrane-bound) with compounds **9-28** and standard inhibitors are shown in Table III.

The following facts should be noted regarding CA inhibition with this type of inhibitors: (i) whereas the mercaptans **9-13** behave as moderate inhibitors against all three isozymes investigated here, their metal complexes act as potent inhibitors, similarly to the unsubstituted sulfonamides **1-3** with clinical applications. The most efficient inhibitors were the Hg(II) complexes, followed by the Cu(I) derivatives and the Zn(II) complexes; (ii) in the series of mercaptans **9-13** as well as for the correponding complexes, the most active

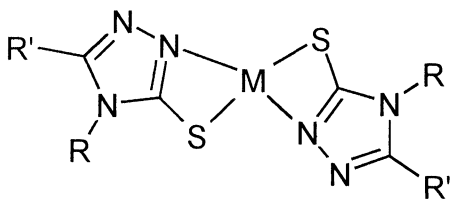

**14-18**: M = Zn
**19-23**: M = Hg

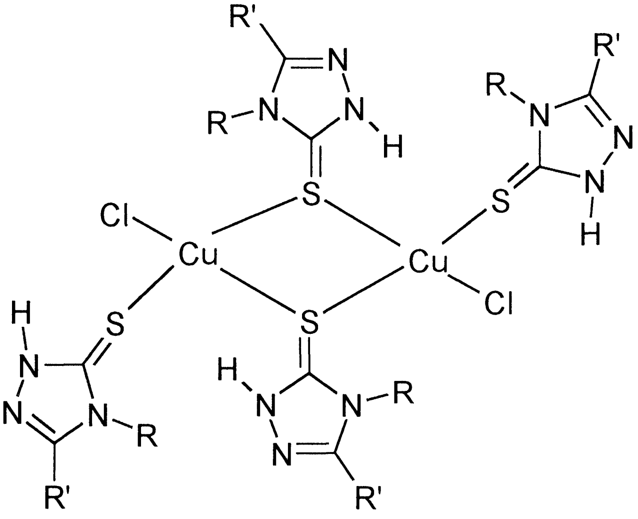

**24-28**

Table III: CA inhibition data with standard inhibitors and compounds **9-28**.

| Compound | $IC_{50}$ (nM)* | | |
|---|---|---|---|
| | hCA I[a] | hCA II[a] | bCA IV[b] |
| **1 (acetazolamide)** | 200±4 | 7±0.2 | 120±9 |
| **2 (benzolamide)** | 10±1 | 2±0.5 | 8±0.3 |
| **3 (ethoxzolamide)** | 8±0.9 | 2±0.2 | 4±0.2 |
| **9** | 1200±60 | 189±5 | 170±6 |
| **10** | 1130±20 | 175±4 | 160±7 |
| **11** | 1040±30 | 162±4 | 160±2 |
| **12** | 1560±45 | 210±8 | 194±10 |
| **13** | 870±12 | 96±8 | 121±11 |
| **14** | 160±2 | 45±3 | 130±5 |
| **15** | 150±3 | 33±2 | 125±3 |
| **16** | 110±2 | 30±1 | 124±8 |
| **17** | 387±5 | 190±0.6 | 245±2 |
| **18** | 67±6 | 15±1 | 27±1 |
| **19** | 12±2 | 3±0.1 | 6±0.7 |
| **20** | 10±0.9 | 3±0.3 | 6±0.9 |
| **21** | 8±0.4 | 2±0.2 | 4±0.2 |
| **22** | 230±8 | 150±9 | 180±5 |
| **23** | 3±0.3 | 0.5±0.1 | 3±0.1 |
| **24** | 16±1 | 5±0.1 | 7±0.1 |
| **25** | 14±1 | 4±1 | 9±0.2 |
| **26** | 6±1 | 4±0.7 | 5±0.6 |
| **27** | 307±5 | 167±12 | 215±10 |
| **28** | 4±0.1 | 0.8±0.1 | 1±0.1 |

* Mean ± average spread (from two determinations).
[a]Human (cloned) isozyme; [b]Isolated from bovine lung microsomes.

inhibitors were those possessing an N-Et or NH moiety in the 4 position of the heterocyclic ring, whereas the compounds possessing N-cyclohexyl such groups had a largely decreased affinity for the enzyme, presumably due to steric hindrance induced by the bulky cyclohexyl group; (iii) the susceptibility of the different CA isozymes to inhibition with this class of derivatives was: CA II > CA IV > CA I, being similar to that for the aromatic/heterocyclic sulfonamides [4].

The newly prepared complexes as well as the heterocyclic mercaptans **9-13** were tested for their ability to lower intraocular pressure (IOP) in rabbits, in animal models of glaucoma, since recently it was discovered by this group that metal complexes of heterocyclic sulfonamides act as efficient IOP lowering agents [48-49]. None of these derivatives showed any effect when applyed as a 2% solution (in DMSO) directly into the rabbit eye.

**Acknowledgments**. CTS is extremely grateful to Professor Eric S. Raper ((University of Northumbria at Newcastle, England) for helpful discussions and suggestions in the field of synthesis of metal complexes of heterocyclic mercaptans. This research was financed in part by a Roumanian Academy grant.

**References**

1 Preceding part of this series: Scozzafava A, Supuran CT (1997) *Metal Based Drugs,* **4**, 19-26.
2 Hewett-Emmett D, Tashian RE (1996) *Mol Phylogenet Evol* **5**, 50-77.
3 Henry RP (1996) *Annu Rev Physiol* **58**, 523-538.
4 Supuran CT (1994) "Carbonic anhydrase inhibitors", in "Carbonic Anhydrase and Modulation of Physiologic and Pathologic Processes in the Organism", (Puscas I, Ed) Helicon, Timisoara, pp. 29-111.
5 Winkler CA, Kittelberger AM, Schwartz GJ (1997) *Am J Physiol* **272**, F551-F560.
6 Maren TH, Conroy CW, Wynns GC, Godman DR (1997) *J Pharmacol Exp Ther* **280**, 98-104.
7 Dodgson SJ, Cherian K (1990) *Arch Biochem Biophys* **282**, 1-7.
8 Dodgson SJ (1987) *J Appl Physiol* **63**, 2134-2141.
9 Hazen SA, Waheed A, Sly WS, LaNoue KF, Lynch CJ (1996) *FASEB J* **10**, 481-490.
10 Supuran CT, Conroy CW, Maren TH (1996) *Eur J Med Chem* **31**, 843-846.
11 Supuran, CT, Nicolae, A, Popescu, A (1996) *Eur J Med Chem* **31**, 431-438.
12 Supuran, CT, Popescu, A, Ilisiu, M, Costandache, A, Banciu, MD (1996) *Eur J Med Chem* **31**, 439- 447.
13 Supuran, CT, Clare, BW (1995) *Eur J Med Chem* **30**, 687-696.
14 a) Supuran CT, Scozzafava A (1997) *J Enzyme Inhib* **12**, 37-51; b) Supuran CT, Briganti F., Scozzafava A (1997) *J Enzyme Inhib* **12**, 175-190.
15 Briganti F, Pierattelli R, Scozzafava A, Supuran CT (1996) *Eur J Med Chem* **31**, 1001- 1010.
16 Supuran CT, Scozzafava A, Popescu A, Bobes-Tureac R, Banciu A, Creanga A, Bobes-Tureac G, Banciu MD (1997) *Eur J Med Chem* **32**, 445-452.
17 Bertini I, Briganti F, Scozzafava A (1995) Zinc proteins. In *Handbook of Metal - Ligand Interactions of Biological Fluids* (Vol. 1), (Berthon G ed), M Dekker, New York, pp 175-191.
18 Bertini I, Canti G, Luchinat C, Scozzafava A (1978) *J Am Chem Soc* **100**, 4873-4877.
19 Bertini I, Luchinat C, Scozzafava A (1982) *Struct Bonding* **48**, 45-92.
20 Tu C, Paranawithana SR, Jewell DA, Tanhauser SM, LoGrasso PV, Wynns GC, Laipis PJ, Silverman DN (1990) *Biochemistry* **29**, 6400-6405
21 Puscas I, Supuran CT (1996) "Farmacologia clinica da ulcer peptica". In *Aparelho Digestivo*, (Coelho, J ed), Rio de Janeiro, MEDSI, 1704-1734.
22 Maren TH (1991) "The links among biochemistry, physiology and pharmacology in carbonic anhydrase mediated systems". In *Carbonic Anhydrase - From Biochemistry and Genetics to Physiology and Clinical Medicine*, (Botré F, Gros G, Storey BT Eds) VCH, Weinheim, pp. 186-207.
23 Bayer A, Ferrari F, Maren TH, Erb C (1996) *J Fr Ophtalmol* **19**, 357-362.
24 Sugrue MF (1996) *J Ocul Pharmacol Ther* **12**, 363-376.
25 Weiner IM (1990) "Diuretics and other agents employed in the mobilization of edema fluids". In *The Pharmacological Basis of Therapeutics*, 8th Edition, Gilman AG, Rall TW, Nies AS, Taylor P Eds, Pergamon Press, New York, pp 713-732.
26 Reiss WG, Oles KS (1996) *Ann Pharmacother* **30**, 514-519.
27 Stoll M, Hamann GF, Jost V, Bompotti UA, Fitridge R, Schimrigk K (1996) *J Neuroimaging* **6**, 144-149.
28 Levine RL, Tursky PA, Turnipseed WD, Grist T (1996) *J Neuroimaging* **6**, 126-130.
29 Supuran CT, Manole G, Dinculescu A, Schiketanz A, Gheorghiu MD, Puscas I, Balaban AT (1992) *J Pharm Sci* **81**, 716-719.

30 Clare BW, Supuran CT (1997) *Eur J Med Chem* **32**, 311-319.
31 Timotheatou D, Ioannou PV, Scozzafava A, Briganti F, Supuran CT (1996) *Metal Based Drugs* **3**, 263-268.
32 Supuran CT, Serves SV, Ioannou PV (1996) *J Inorg Biochem* **62**, 207-212.
33 Supuran CT, Muresan V, Popescu R, Fenesan I (1995) *Main Group Met Chem* **18**, 629-632.
34 Supuran CT, Saramet I, Banciu MD (1995) *Rev Roum Chim* **40**, 1227-1232.
35 Scolnick LR, Clements AM, Liao J, Crenshaw L, Hellberg M, May J, Dean TR, Christianson DW (1997) *J Am Chem Soc* **119**, 850-851.
36 Supuran CT, Banciu MD, Botez G, Balaban AT (1992) *Rev Roum Chim* **37**, 1375-1383.
37 Drago RS (1977) in "Physical Methods in Chemistry", W.B. Saunders & Co., London, p. 411.
38 a) Saramet I, Banciu MD, Draghici C (1991) *Rev Roum Chim* **36**, 127-134; b) Saramet I, Banciu MD, Draghici C (1991) *Rev Roum Chim* **36**, 135-143.
39 .a) Forsman C, Behravan G, Osterman A, Jonsson BH (1988) *Acta Chem Scand* **B42**, 314-318; b) Behravan G, Jonasson P, Jonsson BH, Lindskog S (1991), *Eur J Biochem* **198**, 589-592.
40 Khalifah RG, Strader DJ, Bryant SH, Gibson SM (1977) *Biochemistry* **16**, 2241-2247.
41 a) Nyman PO, Lindskog S (1964) *Biochim Biophys Acta* **85**, 141-151; b) Henderson LE, Henriksson D, Nyman PO (1976) *J Biol Chem* **251**, 5457-5463.
42 Maren TH, Wynns GC, Wistrand PJ (1993) *Mol Pharmacol* **44**, 901-906
43 Pocker Y, Stone JT (1967) *Biochemistry* **6**, 668-679.
44 a) Raper ES (1994) *Coord Chem Rev* **129**, 91-156; b) Raper ES (1996) *Coord Chem Rev* **153**, 199-255; c) Raper ES (1997) *Coord Chem Rev* in press.
45 a) Creighton JR, Gardiner DJ, Gorvin AC, Gutteridge C, Jackson ARW, Raper ES, Sherwood PMA (1985) *Inorg Chim Acta* **103**, 195-205; b) Oughtred RE, Raper ES, Nowell IW (1985) *Acta Crystallogr C*, **41**, 758; c) Atkinson ER, Gardiner DJ, Jackson ARW, Raper ES (1985) *Inorg Chim Acta* **98**, 35-41.
46 a) Atkinson ER, Raper ES, Gardiner DJ, Dawes HM, Walker NPC, Jackson ARW, (1985) *Inorg Chim Acta* **100**, 285-291; b) Raper ES, Creighton JR, Wilson JD, Clegg W, Milne A (1988) *Inorg Chim Acta* **149**, 265-271; c) Raper ES, Wilson JD, Clegg W (1992) *Inorg Chim Acta* **194**, 51-55.
47 a) Bigoli F, Lanfranchi M, Pellinghelli MA (1990) *J Chem Res* 214-215; b) Cingi MB, Bigoli F, Lanfranchi M, Pellinghelli MA, Vera A, Buluggiu E (1992) *J Chem Soc Dalton Trans* 3145-3151.
48 Supuran CT, Scozzafava A, Saramet I, Banciu MD (1997) *J Enzyme Inhib*, in press.
49 Supuran CT, Scozzafava A, Jitianu A (1997) *Metal Based Drugs*, in press.

**Received: October 4, 1997 - Accepted: December 3, 1997 - Received in revised camera-ready format: December 9, 1997**

